# Alien plant species distribution in Romania: a nationwide survey following the implementation of the EU Regulation on Invasive Alien Species

**DOI:** 10.3897/BDJ.12.e119539

**Published:** 2024-05-28

**Authors:** Paulina Anastasiu, Iulia V Miu, Athanasios A Gavrilidis, Cristina Preda, Laurentiu Rozylowicz, Culita Sirbu, Adrian Oprea, Mihaela Urziceanu, Petronela Camen-Comanescu, Eugenia Nagoda, Daniyar Memedemin, Marius Barbos, Violeta Boruz, Alina Cislariu, Ioan Don, Marius Fagaras, Jozsef Pal Frink, Ioana Mihaela Georgescu, Ovidiu Ioan Haruta, Bogdan-Iuliu Hurdu, Attila Matis, Sretco Milanovici, Sorana Muncaciu, Alina Georgeta Neacsu, Monica Neblea, Alma Lioara Nicolin, Mariana Niculescu, Silvia Oroian, Oliviu Grigore Pop, Daniel I Radutoiu, Mihaela Samarghitan, Ioana Simion, Liliana Cristina Soare, Corina Steiu, Emilia Stoianov, Daniela Strat, Anna Szabo, Paul Marian Szatmari, Corneliu Tanase, Marian D Mirea, Nicolae Manta, Ioana M Sirbu

**Affiliations:** 1 University of Bucharest, Dimitrie Brandza Botanic Garden, Bucharest, Romania University of Bucharest, Dimitrie Brandza Botanic Garden Bucharest Romania; 2 University of Bucharest, Faculty of Biology, Bucharest, Romania University of Bucharest, Faculty of Biology Bucharest Romania; 3 University of Bucharest, Center for Environmental Research, Bucharest, Romania University of Bucharest, Center for Environmental Research Bucharest Romania; 4 Ovidius University of Constanta, Faculty of Natural and Agricultural Sciences, Constanta, Romania Ovidius University of Constanta, Faculty of Natural and Agricultural Sciences Constanta Romania; 5 University of Agricultural Sciences and Veterinary Medicine Ion Ionescu de la Brad, Faculty of Agriculture, Iasi, Romania University of Agricultural Sciences and Veterinary Medicine Ion Ionescu de la Brad, Faculty of Agriculture Iasi Romania; 6 Alexandru Ioan Cuza University of Iasi, Faculty of Biology, Iasi, Romania Alexandru Ioan Cuza University of Iasi, Faculty of Biology Iasi Romania; 7 Alexandru Ioan Cuza University of Iasi, Anastasie Fatu Botanic Garden, Iasi, Romania Alexandru Ioan Cuza University of Iasi, Anastasie Fatu Botanic Garden Iasi Romania; 8 University of Bucharest, Research Institute of University of Bucharest, Bucharest, Romania University of Bucharest, Research Institute of University of Bucharest Bucharest Romania; 9 Chelonia Romania, Bucharest, Romania Chelonia Romania Bucharest Romania; 10 GTM CO SRL, Cluj-Napoca, Romania GTM CO SRL Cluj-Napoca Romania; 11 University of Craiova, Alexandru Buia Botanic Garden, Craiova, Romania University of Craiova, Alexandru Buia Botanic Garden Craiova Romania; 12 Vasile Goldis Western University of Arad, Arad, Romania Vasile Goldis Western University of Arad Arad Romania; 13 National Institute for Research and Development in Forestry Marin Dracea, Cluj-Napoca, Romania National Institute for Research and Development in Forestry Marin Dracea Cluj-Napoca Romania; 14 University of Agricultural Sciences and Veterinary Medicine, Bucharest, Romania University of Agricultural Sciences and Veterinary Medicine Bucharest Romania; 15 University of Oradea, Department of Forestry and Forestry Engineering, Oradea, Romania University of Oradea, Department of Forestry and Forestry Engineering Oradea Romania; 16 Institute of Biological Research Cluj-Napoca, Cluj-Napoca, Romania Institute of Biological Research Cluj-Napoca Cluj-Napoca Romania; 17 Babes-Bolyai University Cluj-Napoca, Faculty of Biology and Geology, Cluj-Napoca, Romania Babes-Bolyai University Cluj-Napoca, Faculty of Biology and Geology Cluj-Napoca Romania; 18 Romsilva Cheile Nerei Beusnita National Park Administration, Sasca Montana, Romania Romsilva Cheile Nerei Beusnita National Park Administration Sasca Montana Romania; 19 King Mihai I University of Life Sciences of Timisoara, Timisoara, Romania King Mihai I University of Life Sciences of Timisoara Timisoara Romania; 20 National University of Science and Technology - Politehnica Bucharest - Pitesti University Center, Pitesti, Romania National University of Science and Technology - Politehnica Bucharest - Pitesti University Center Pitesti Romania; 21 myNature Association, Timisoara, Romania myNature Association Timisoara Romania; 22 University of Craiova, Faculty of Agronomy, Department of Botany and Biodiversity Conservation, Craiova, Romania University of Craiova, Faculty of Agronomy, Department of Botany and Biodiversity Conservation Craiova Romania; 23 George Emil Palade University of Medicine, Pharmacy, Sciences and Technology, Targu Mures, Romania George Emil Palade University of Medicine, Pharmacy, Sciences and Technology Targu Mures Romania; 24 Conservation Carpathia Foundation; Renaturopa Association, Brasov, Romania Conservation Carpathia Foundation; Renaturopa Association Brasov Romania; 25 University of Craiova, Faculty of Horticulture, Department of Biology and Environmental Engineering, Craiova, Romania University of Craiova, Faculty of Horticulture, Department of Biology and Environmental Engineering Craiova Romania; 26 Mures County Museum, Natural Sciences Section, Targu Mures, Romania Mures County Museum, Natural Sciences Section Targu Mures Romania; 27 P.P.V.N.C. Excelsior Association, Timisoara, Romania P.P.V.N.C. Excelsior Association Timisoara Romania; 28 University of Bucharest, Faculty of Geography, Bucharest, Romania University of Bucharest, Faculty of Geography Bucharest Romania; 29 Romanian Ornithological Society, Cluj-Napoca, Romania Romanian Ornithological Society Cluj-Napoca Romania; 30 Biological Research Center - Vasile Fati Botanical Garden, Jibou, Romania Biological Research Center - Vasile Fati Botanical Garden Jibou Romania; 31 Romanian Ministry of Environment, Water and Forests, Bucharest, Romania Romanian Ministry of Environment, Water and Forests Bucharest Romania

**Keywords:** invasive plant species, occurrence records, species richness, exotic species, European Union

## Abstract

**Background:**

Biological invasions pose an increasing risk to nature, social security and the economy, being ranked amongst the top five threats to biodiversity. Managing alien and invasive species is a priority for the European Union, as outlined in the EU Biodiversity Strategy for 2030 and the Kunming-Montreal Global Biodiversity Framework. Alien plant species are acknowledged to impact the economy and biodiversity; thus, analysing the distribution of such species provides valuable inputs for the management and decision-making processes. The database presented in the current study is the first consolidated checklist of alien plant species that are present in Romania, both of European Union concern and of national interest. This database complements a prior published distribution, based only on records from literature, bringing new information regarding the occurrence of alien plants in Romania, as revealed by a nationwide field survey. We consider this database a valuable instrument for managing biological invasions at both national and regional levels, as it can be utilised in further research studies and in drafting management and action plans, assisting stakeholders in making informed decisions and implementing management actions.

**New information:**

We present the results of the first nationwide survey of alien plant species in Romania, conducted between 2019 and 2022, in the framework of a national project coordinated by the Ministry of Environment, Waters and Forests and the University of Bucharest. The present database complements and updates the database published by Sirbu et. al (2022), which included occurrence records published until 2019. The new database includes 98323 occurrence records for 396 alien plant species in 77 families, with most species belonging to the Asteraceae family. One alien plant species in our database, the black locust *Robiniapseudoacacia* L., had more than 10,000 occurrence records. The distribution database also includes information on newly-reported invasive alien plant species of European Union concern in Romania (i.e. the floating primrose-willow *Ludwigiapeploides* (Kunth) P.H.Raven) and documents the presence of plants in 44 additional families compared to Sirbu et al. (2022). Each entry includes information on species taxonomy, location, year, person who recorded and identified the alien plant, geographical coordinates and taxon rank.

## Introduction

Biological invasions pose an increasing risk to biodiversity, social security and the economy, resulting in annual impacts amounting to hundreds of billions USD (Zenni et al. 2021, Haubrock et al. 2021) and in ranking amongst the top current and future threats to biodiversity (Seebens et al. 2020). Human activities facilitated the introduction and establishment of more than 37,000 alien species around the world, with approximately 200 new alien species being recorded in each year (Roy et al. 2023). The number of invasive alien species and their impacts are increasing rapidly and are likely to continue rising (Seebens et al. 2017, Seebens et al. 2020, Pyšek et al. 2020, Roy et al. 2023), which is why addressing the issue of biological invasions is of utmost importance (European Commission 2020, CBD 2022).

The management of alien species requires cooperation, robust transnational policy instruments and effective enforcement from states. In the European Union, one of the most important policy instruments and relevant in terms of the management and control of biological invasions is Regulation 1143/2014 of the European Parliament and of the Council of 22 October 2014 on the prevention and management for the introduction and spread of invasive alien species (European Union 2014). The list of invasive alien species of EU concern adopted by the Commission Implementing Regulation in 2016 was subsequently updated and currently includes 88 species, of which 41 are invasive alien plants (European Commission 2022). Following this legal document, EU Member states are required to map the distribution of invasive alien species (hereafter IAS) of EU concern on their territory, to elaborate and implement action plans on the pathways of introduction, to put in place an early detection and rapid eradication system and to implement management actions (Brundu et al. 2022).

Several local and regional studies focused on alien and invasive plant species and raising awareness about biological invasions were published in Romania in the last decades (e.g. Dihoru (2004), Anastasiu and Negrean (2005), Anastasiu and Negrean (2009), Sirbu and Oprea (2011), Anastasiu et al. (2016), Anastasiu et al. (2017), Sirbu et al. (2021), Urziceanu et al. (2024)). Sirbu et al. (2022) collected 42,776 occurrence records of alien plant species from studies published between 1778 and 2018, compiling the first complete national database of alien and invasive plants for Romania (Universitatea din Bucuresti and Ministerul Mediului, Apelor si Padurilor 2023).

Aside from managing alien species that are already present in an area of interest, an important step in reducing the ecological and socioeconomic impacts of alien species is to prevent their introduction (Keller et al. 2011). This can be achieved by horizon-scanning exercises, surveying the area of interest and systematically searching for species found in the neighbourhood. Such information can be provided by, for example, open-access distribution databases (Latombe et al. 2017). These databases are also essential tools for risk assessments and useful for rapid reaction leading to eradication or containment and control (Hulme et al. 2009).

## General description

### Purpose

This study aims to update the known distribution of alien and invasive plant species in Romania published in 2022 by Sirbu et al. (2022), with occurrences collected in the field between 2019 and 2022 during a nationwide survey and to assess the distribution and diversity of alien plant species in Romania, by analysing spatial patterns of distribution data. Despite the urgency of managing biological invasions, current knowledge on the distribution of alien and invasive plant species at the national level is incomplete with large unsampled areas. With this distribution database, we provide up-to-date information that can help stakeholders and policy-makers take steps towards an efficient management of alien and invasive plant species in Romania.

### Additional information

The nationwide field survey took place in the framework of the project Operational Programme for Large Infrastructure (POIM 2014+ 120008) Invasive species management in Romania according to REGULATION (EU) 1143/2014 on the prevention and management of the introduction and spread of invasive alien species (European Union 2014). The project aimed to inventory and map the occurrence of alien species in Romania, create a legally binding action plan to address the priority pathways of introduction and implement a comprehensive awareness-raising campaign (Universitatea din Bucuresti and Ministerul Mediului, Apelor si Padurilor 2023).

## Project description

### Study area description

Romania is situated in the central part of Europe and lies between latitudes 43º and 49º N and longitudes 20º and 30º E. The country's surface is 238,397 km^2^. Romania has a varied relief landscape that encompasses the Carpathian Mountains, which are the dominant mountain range in Romania with the highest altitude at 2544 m, sub-Carpathian hills, plateaus, plains and the Danube Delta. The Danube, Europe's second-longest river, flows through the southern part of Romania. Romania's eastern border is defined by the Black Sea (Rey et al. 2007). Romania overlaps five terrestrial biogeographical regions out of the nine regions recognised by the European Union, i.e. Alpine, Continental, Pannonian, Steppic and Black Sea (Rozylowicz et al. 2019). Due to its varied geography, Romania has a temperate-continental climate with sub-Mediterranean influences in the south and southeast, continental influences in the central and eastern part and Atlantic influences in the west (Rey et al. 2007). A variety of habitat types are present in Romania, from coastal and halophytic habitats, sea dunes, freshwater habitats, temperate heath and scrub, natural and semi-natural grasslands, bogs, rocky habitats and caves, coniferous and deciduous forests (Ciocarlan 2009).

### Design description

A nationwide survey took place over a period of 4 years, between 2019 and 2022. The observations were conducted each year during the optimal months for the development of alien species (June-November). A total of 98,323 occurrence records belonging to 396 alien plant species were recorded during the survey, including the species of EU concern. In the datasets, every entry is linked with taxonomic details, such as species, genus, family, order, class, phylum, kingdom, infraspecific epithet, taxon rank, as well as information regarding the sampling event, including date, location and geographical coordinates. Analysing the spatial distribution of alien plant species will lead in designing an effective management, as well as highlighting hotspots characterised by the presence of a high number of alien species or where occurrences of species EU concern have been reported. The identified hotspots can serve as a complementary method, useful in identifying priority introduction pathways.

## Sampling methods

### Sampling description

Two survey methods were used: low-intensity survey and high-intensity survey. For the low-intensity survey, the survey of alien plant species was carried out in randomly selected grids of 100 km^2^ to cover Romania's surface in a balanced manner. Botanists randomly selected 624 transects that were 50 km long and up to 50 m in width crossing over five consecutive 100 km^2^ quadrats, with a sampling station at each 10 km (i.e. five sampling stations on each transect). In the case of high-intensity surveys, the survey was carried out in 18,654 quadrats of 100 m^2^ quadrats located in randomly selected areas in every county. Occurrence data were collected with a GPS and survey forms were completed alongside photos for habitat and species identification. Specimens posing difficulty in on-site identification were collected for further examination and identification in the lab. Database curation was carried out by experienced botanists and biogeographers (authors of the paper).

## Geographic coverage

### Description

Romania

### Coordinates

43.68844 and 48.22088 Latitude; 20.22019 and 29.62654 Longitude.

## Taxonomic coverage

### Description

A total of 98,323 occurrence records belonging to 396 alien plant species were documented, covering the entire country. The documented alien plant species cover 77 families, with most species belonging to Asteraceae (16.7%), followed by Poaceae (6.8%), Amaranthaceae (6.3%), Fabaceae (5.8%) and Rosaceae (5%) families (Table 1). A total of 28 families comprise only one species each and the families Buxaceae, Cleomaceae, Linaceae, Menispermaceae, Musaceae, Nelumbonaceae, Pinaceae, Pteridaceae, Rhamnaceae and Rubiaceae had only one species and one occurrence record per species. Amongst the 396 specific taxa, six are infraspecific, of which four subspecies (i.e. Xanthiumorientalesubsp.italicum (Moretti) Greuter, OenotheramacrocarpaNutt.subsp.macrocarpa, OenotheravillosaThunb.subsp.villosa and Solanumtriflorumsubsp.ponticum (Prodan) Borza and two varieties (i.e. Atriplexhortensisvar.rubra L. and Violasororiavar.priceana (Pollar) Coopper. (Suppl. material 1). The species taxonomy considered in the present paper is based on the GBIF Backbone Taxonomy (GBIF Secretariat 2023).

The species with the most records (i.e. over 10,000 occurrences) is the black locust *Robiniapseudoacacia* L., followed by the ragweed *Ambrosiaartemisiifolia* L., the daisy fleabane *Erigeronannuus* (L.) Pers., the horseweed *Erigeroncanadensis* L. and Xanthiumorientalesubsp.italicum, with more than 5000 occurrence records each, while 212 alien plant species had less than ten occurrence records per species (Table 1). *Ambrosiaartemisiifolia* and *Erigeroncanadensis* also had the largest number of occurrences reported in the list published by Sirbu et al. (2022).

The distribution database documents the presence of seven invasive alien plant species of EU concern in Romania (European Commission 2022), i.e. *Ailanthusaltissima* (Mill.) Swingle, *Asclepiassyriaca* L., *Elodeanuttallii* (Planch.) H.St.John, *Heracleumsosnowskyi* Manden., *Humulusscandens* (Lour.) Merr., *Impatiensglandulifera* Royle and *Ludwigiapeploides* (Kunth) P.H.Raven. Compared with the alien and invasive plant species list published by Sirbu et al. (2022), the presence of *Cabombacaroliniana* A.Gray and *Myriophyllumaquaticum* (Vell.) Verdc. in Romania was not confirmed. *Heracleumsosnowskyi*, which had only one record in the list published by *Sirbu et al. (2022)*, was confirmed with 12 more occurrence records from the same area in the central part of Romania. The presence of *Ludwigiapeploides* was observed for the first time by Sirbu et al. (2021) in the southern part of Romania. Presently, there are only four reports of its occurrence.

## Usage licence

### Usage licence

Creative Commons Public Domain Waiver (CC-Zero)

### IP rights notes

This work is licensed under the Creative Commons Attribution 4.0 International Licence. To view a copy of this licence, visit http://creativecommons.org/ licenses/by/4.0/.

## Data resources

### Data package title

Distribution of alien plant species in Romania as resulted from the nationwide survey conducted between 2019 and 2022.

### Resource link

https://doi.org/10.15468/zk9adu

### Alternative identifiers

https://ipt.pensoft.net/resource?r=alien_plant_ro

### Number of data sets

1

### Data set 1.

#### Data set name

alien_plant_ro

#### Data format

Darwin Core Archive format

#### Character set

UTF-8

#### Download URL

https://doi.org/10.15468/zk9adu

#### Description

The database was published in the Global Biodiversity Information Facility platform, GBIF. The database comprises information about alien plant species in Romania. The database includes 98,323 occurrence records belonging to 396 alien plant species. The database submitted to GBIF is structured as a sample event dataset that has been published as a Darwin Core Archive (DwCA), which is a standardised format for sharing biodiversity data as a set of one or more data tables. A description of the column headers used is given below. The Darwin Core Standard (DwC) was used to offer a "stable, straightforward and flexible framework for compiling biodiversity data from varied and variable sources" (https://www.gbif.org/en/darwin-core). All column labels and descriptions are from https://dwc.tdwg.org/terms/.

**Data set 1.** 

Column label	Column description
occurrenceID	An identifier of a particular occurrence unique within this dataset. We used a combination of the organisation's abbreviation and numbers; http://rs.tdwg.org/dwc/terms/occurrenceID
institutionCode	The name (or acronym) in use by the institution having custody of the object(s) or information referred to in the record; http://rs.tdwg.org/dwc/terms/institutionCode
recordedBy	A person or group of people who were the primary observer; http://rs.tdwg.org/dwc/iri/recordedBy
basisOfRecord	The specific nature of the data record. Included value: HumanObservation;http://rs.tdwg.org/dwc/terms/basisOfRecord
identifiedBy	A list of names of people who assigned the Taxon to the subject; http://rs.tdwg.org/dwc/iri/identifiedBy
continent	One value – Europe;http://rs.tdwg.org/dwc/terms/continent
country	One value – Romania;http://rs.tdwg.org/dwc/terms/country
countryCode	One value – RO;http://rs.tdwg.org/dwc/terms/countryCode
county	The full, unabbreviated name of the next smaller administrative region than stateProvince (county) in which the dcterms:Location occurs;http://rs.tdwg.org/dwc/terms/county
locality	The specific description of the place (in Romanian); http://rs.tdwg.org/dwc/terms/locality
kingdom	The full scientific name of the kingdom in which the taxon is classified;http://rs.tdwg.org/dwc/terms/kingdom
phylum	The full scientific name of the phylum or division in which the taxon is classified;http://rs.tdwg.org/dwc/terms/phylum
class	The full scientific name of the class in which the taxon is classified; http://rs.tdwg.org/dwc/terms/class
order	The full scientific name of the order in which the taxon is classified; http://rs.tdwg.org/dwc/terms/order
family	The full scientific name of the family in which the taxon is classified; http://rs.tdwg.org/dwc/terms/family
Genus	The full scientific name of the genus in which the taxon is classified; http://rs.tdwg.org/dwc/terms/genus
scientificName	The original scientific name; http://rs.tdwg.org/dwc/terms/scientificName
infraspecificEpithet	The name of the lowest or terminal infraspecific epithet of the scientificName, excluding any rank designation; http://rs.tdwg.org/dwc/terms/infraspecificEpithet
taxonRank	The taxonomic rank of the most specific name in the scientificName; http://rs.tdwg.org/dwc/terms/taxonRank
occurrenceStatus	A statement about the presence or absence of a dwc:Taxon at a dcterms:Location;http://rs.tdwg.org/dwc/terms/occurrenceStatus
decimalLongitude	The geographic longitude in decimal degrees; http://rs.tdwg.org/dwc/terms/decimalLongitude
decimalLatitude	The geographic latitude in decimal degrees; http://rs.tdwg.org/dwc/terms/decimalLatitude
geodeticDatum	The ellipsoid, geodetic datum or spatial reference system (SRS) upon which the geographic coordinates given in decimalLatitude and decimalLongitude are based; http://rs.tdwg.org/dwc/iri/geodeticDatum
eventDate	The four-digit year in which the Event occurred, according to the Common Era Calendar;http://rs.tdwg.org/dwc/terms/year

## Additional information

In this article, we also conducted a spatial analysis of alien plant species diversity and distribution, while highlighting areas with high concentrations of alien species. The analysis helps in understanding the spread and extent of alien plant species and identifying potential pathways of introduction and areas vulnerable to invasion. By identifying areas with high alien plant species diversity and distribution, appropriate management strategies can be implemented to control their spread and mitigate their negative effects.

We analysed the spatial patterns of alien plant species occurrences per 5 km × 5 km grid cell at the national level using Global Moran's I test to evaluate the overall spatial pattern of occurrences by indicating if reported occurrences at grid cell level are significantly clustered across Romania (Fortin and Dale 2005) and Getis Ord Gi* spatial statistic to assess the local patterns of sampling bias (Ord and Getis 2010) which identifies clusters of records with values numerically higher than expected by random chance. We identified clusters of UTM 5 km × 5 km cells where the sampling effort was significantly higher (hotspots of occurrence, GiZScore > 1.96) or lower (cold spots of occurrence, GiZScore < 1.96). To compare the number of alien plant species within the taxonomic group at different locations, we assessed their distribution and mapped the species richness. Valid occurrence records were aggregated at a Universal Traverse Mercator (EPSG 9807) spatial resolution of 25 km^2^ (UTM 5 km × 5 km). We also mapped alien plant species richness at a spatial resolution of 50 km × 50 km UTM grid cells. Aggregating species richness at a coarser resolution reduced the potential bias in sampling effort and allowed for a better understanding and visualisation of regional patterns (Graham and Hijmans 2006). Georeferenced data points were transferred to ArcGIS Pro (ESRI, Redlands CA) and visually inspected for errors.

### Results

**Figure 1.**
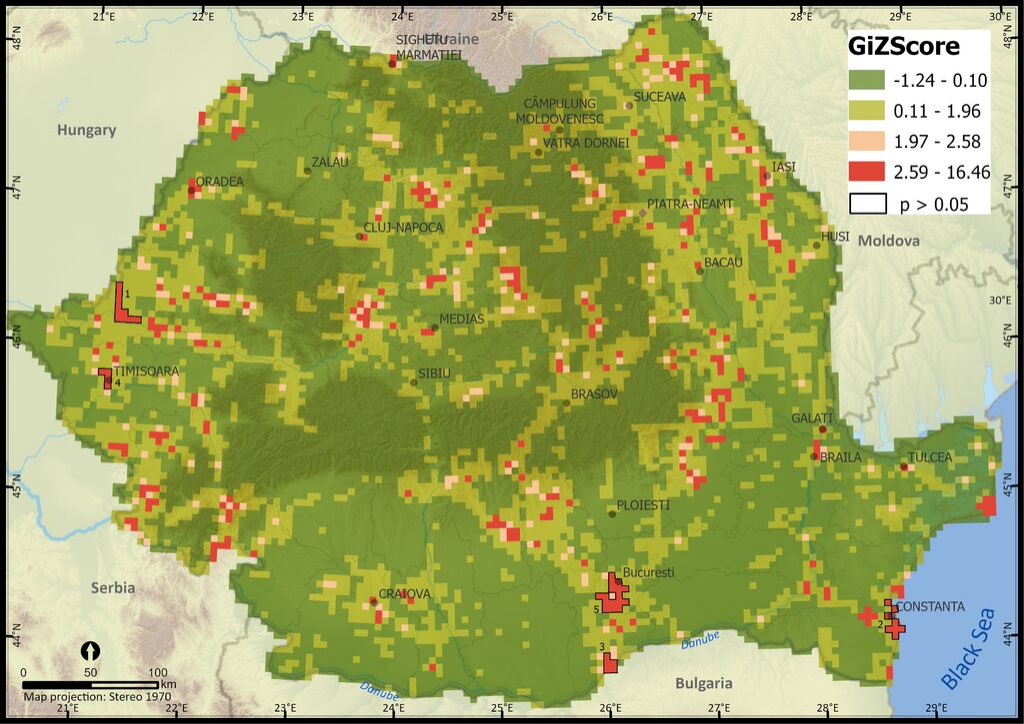
Hotspots of alien plant species survey in Romania (in red) suggesting a significantly clustered pattern in the number of alien plant species occurrences per UTM 5 km × 5 km grid cell. The numbered statistically significant hotspots are: **1** Arad City, **2** Constanta City, **3** Giurgiu City, **4** Timisoara City, **5** Bucharest City.

At the national scale, Global Moran's I test indicated a significantly clustered pattern in the number of alien plant species (Z = 56.54, p < 0.001) and of species occurrences (Z > 1.96, p < 0.05) per UTM 5 km × 5 km grid cell, thus suggesting a strong bias in the distribution of alien plant species. Moreover, results of the Getis Ord Gi* spatial statistic revealed several hotspots of recorded alien plant species. We observed high clusters of records in cities and surroundings with the highest sampling effort recorded in Arad City (mean Z = 7.10) **(1)** and Timisoara (mean Z = 5.04) **(4)** in western Romania, Constanta (mean Z = 6.29) **(2)** in eastern Romania, Giurgiu (mean Z = 6.24) **(3)** and Bucharest (mean Z = 5.00) **(5)** in southern Romania. Moreover, there are several smaller hotspots in Cluj and Alba-Iulia Counties (in the central part of Romania), in Botosani and Iasi Counties (the north-eastern part of Romania) and around the Cities of Oradea (in the western part of Romania) and Sulina (south-eastern Romania) (Fig. 1).

**Figure 2.**
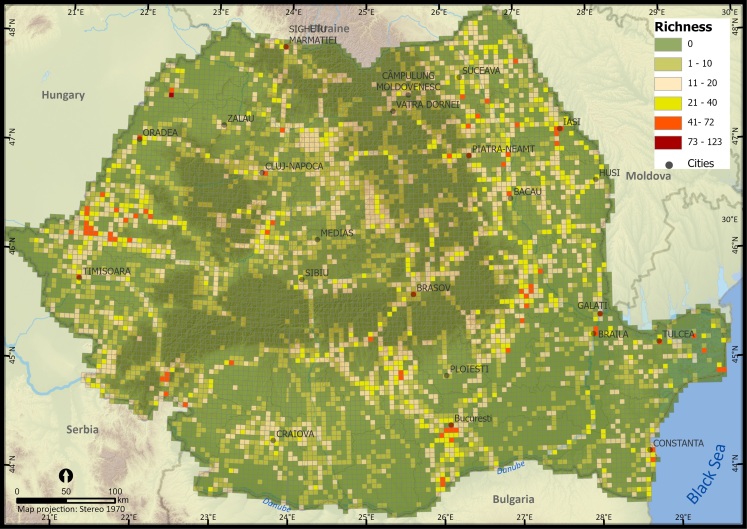
Alien plant species richness in Romania (UTM 5 km × 5 km grid resolution).

Species richness aggregated at a 5 km × 5 km grid ranged from 3 to 123 species. The highest number of alien plant species was recorded in the western part of the country and around cities, namely Oradea and surrounding areas with 123 species per grid cell, followed by Arad with 72 species per grid cell, Timisoara with 55 species per grid cell, in the eastern part of the country, Braila with 51 species per grid cell, Tulcea with 50 species per grid cell and Bucharest in the southern part of the country with 59 species per grid cell. Most of the grid cells with high alien plant species richness recorded are concentrated in particular regions of Romania, i.e. the western part (e.g. Timisoara and Arad Counties), the eastern (e.g. Iasi, Neamt and Vrancea Counties), south-eastern (e.g. Braila, Tulcea and Constanta Counties) and the southern part of Romania (e.g. Bucharest), suggesting a distribution of alien plant species around urban centres and traffic routes (Fig. 2). Grid cells with low richness values are mostly distributed in the southern and northern parts of the country, reflecting an undersampling of alien plant species.

**Figure 3.**
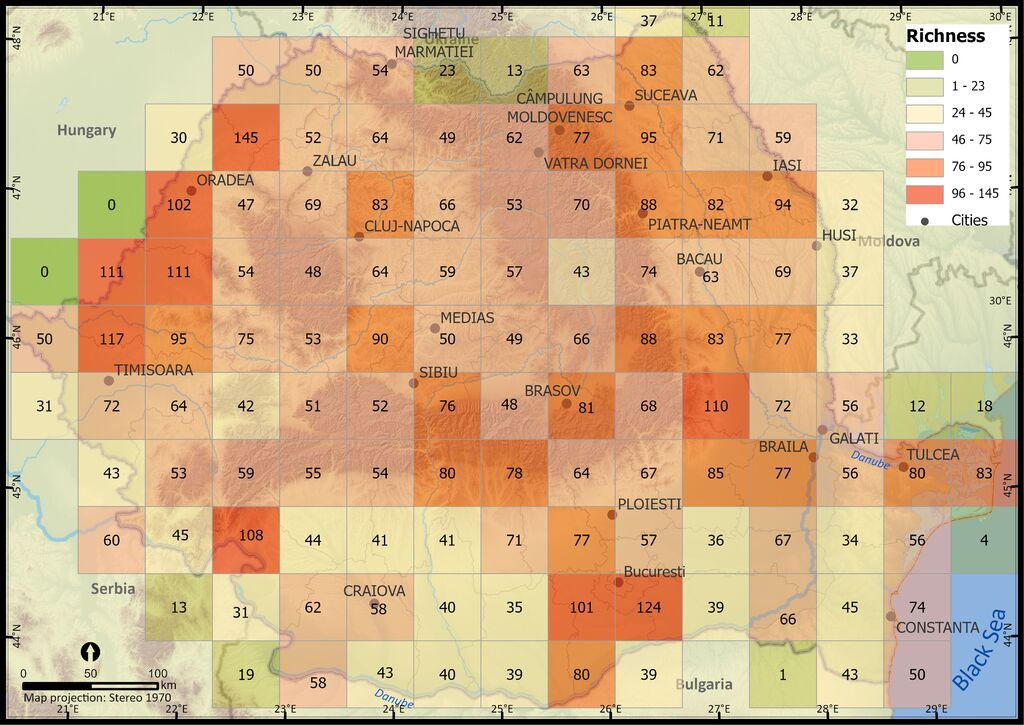
Alien plant species richness in Romania (UTM 50 km × 50 km grid resolution).

When represented at a lower spatial resolution (50 km × 50 km), alien plant species richness ranged from 23 to 145 species per grid cell (Fig. 3). The same patterns can be observed on the map: higher species richness in the western and eastern part of the country and around major cities and the capital (e.g. Oradea, Timisoara, Arad, Iasi, Tulcea, Bucharest) and lower alien plant species richness in the southern and northern parts of Romania.

### Conclusion

The distribution of data collected suggests a correlation between the presence of transportation infrastructure and the occurrence of alien species (Mortensen et al. 2017, Rauschert et al. 2017), as most of the 5 km × 5 km grid cells in which alien plant species were recorded are crossed by, or have roads with high traffic volumes nearby (e.g. European routes E85, E81 and E60) or the cities with the highest number of alien plant species are located close to the borders (i.e. Oradea, Arad, Timisoara, Iasi, Braila, Oradea, Tulcea, Constanta). However, it is important to note that this distribution might be influenced by biases, as the in situ observations relied on the accessibility of the sample plots. This hypothesis serves as a a starting point for future research studies. A similar study by Lemke et al. (2018) demonstrated that traffic volume significantly affected dispersal distances and the lateral deposition of seeds of *Ambrosiaartemisiifolia* on roadsides in Germany. The same invasive alien plant species with a highly allergenic effect on human health was repeatedly reported in Romania, being proposed as a species of interest for Romania and included in the Action plan on the pathways of invasive alien species (Ministerul Mediului, Apelor si Padurilor 2022).

Presently, of the 41 plant species of EU concern, four are already established in Romania and widespread: *Ailanthusaltissima*, *Asclepiassyriaca*, *Elodeanuttallii* and *Impatiensglandulifera*. The presence of *Humulusscandens* was also confirmed in several regions (e.g. southern and southwest, central and northwest parts of the country). *Heracleumsosnowskyi* and *Ludwigiapeploides* are confirmed in one location for each species. The presence of *Cabombacaroliniana* and *Myriophyllumaquaticum* in Romania is not confirmed.

Amongst the 396 alien plant species inventoried in this study, seven species are proposed as species of interest for Romania (i.e. *Ambrosiaartemisiifolia*, *Ambrosiatenuifolia*, *Ambrosiatrifida*, *Cyclachaenaxanthiifolia*, *Phytolaccaamericana*, *Phytolaccaacinosa* and *Verbesinaencelioides*) according to the Article 12(1) of Regulation 1143/2014 of the European Union (EU), where Member States may establish a national list of invasive alien species of interest to a Member State (European Union 2014). The seven alien plant species were selected, based on their significant impact on both human health, such as allergenic pollen and agriculture, particularly in terms of seed production (Lommen et al. 2017, Kumar Rai and Singh 2020). Three of them are also included in the Action plan on the pathways of invasive alien species (i.e. *Ambrosiaartemisiifolia*, *Ambrosiatrifida* and *Phytolaccaamericana*). This indicates that these species are considered to have a significant impact and require special measures for their management.

**Figure 4.**
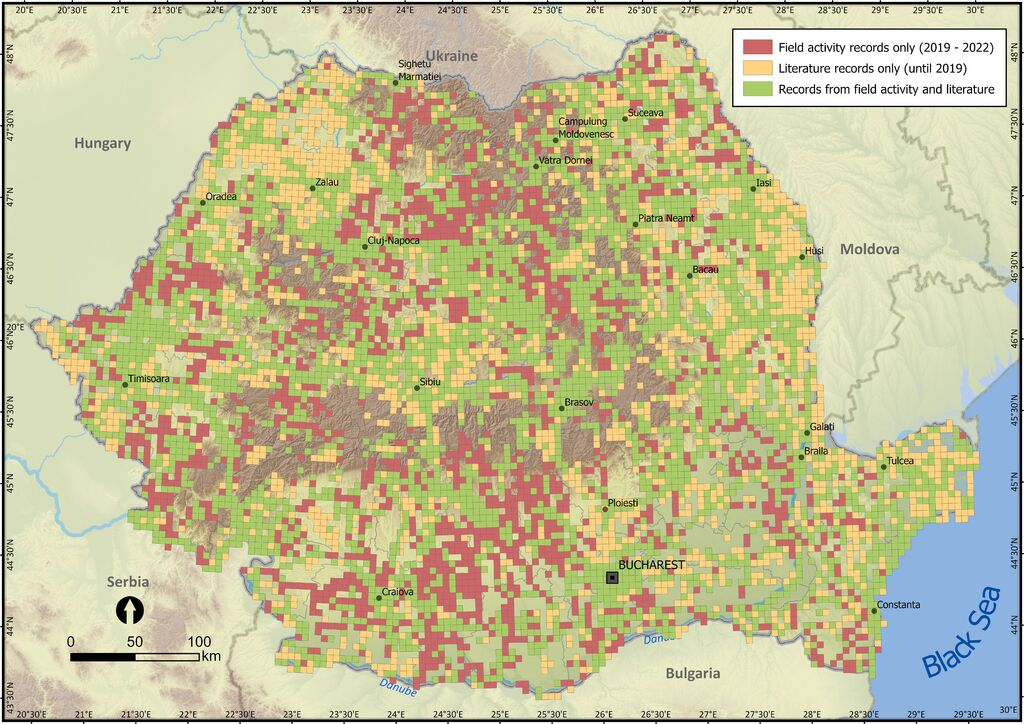
Alien plant species records in Romania, resulting from joining Sirbu et al. (2022) review of literature (1778-2018) and our field survey (2019-2022) at UTM 5 km × 5 km grid resolution.

When comparing with the dataset published by Sirbu et al. (2022) by overlaying the spatially corresponding databases using GIS, the new distribution database shows a more reduced survey bias (Fig. 1). This suggests that our database included in this article reflect better the distribution of alien species and the pathways of introduction. Sirbu et al. (2022) indicate hotspots of invasive alien plant species near major academic and research facilities, concluding that the data collection was opportunistic rather than systematic (see literature records only, grid cells in yellow, Fig. 4). This finding validates the earlier mentioned studies, with this phenomenon attributed to the botanist effect (Moerman and Estabrook 2006). The globalisation era brought the spotlight upon transport networks, which are considered the frontline in the prevention of biological invasions (Hulme et al. 2009). Our results showed that most of the 5 km × 5 km cells in which alien plant species were recorded are crossed by important roads (high volumes of traffic roads, see field activity records only, grid cells in red, Fig. 4). This outcome endorses the prior studies acknowledging the role of transport infrastructures in the dispersal of alien plant species (Rauschert et al. 2017).

In the field inventory-based database, we noted limited coverage of the northern and southern parts of Romania, facts also noticed by Sirbu et al. (2022) in the literature-based database. We also observed that high species richness matches the hotspots of sampling efforts, validating the finding that sampling in our survey was directly correlated with alien plant species diversity and ease of access (Fig. 4). The analysis revealed that high species richness was observed in the same areas mentioned by Sirbu et. al (2002) in his study (e.g. western, eastern, central and south-eastern parts of the country, as well as near major cities such as Arad, Timisoara, Oradea, Bucharest, Constanta and Iasi), indicating that urban environments frequently serve as entry points for alien species (Botham et al. 2009) (see records from field activity and literature, grid cells in green, Fig. 4). Thus, referencing bias in sampling serves to acknowledge the potential limitations in the comprehensiveness and representativeness of the collected data, despite efforts to achieve national coverage.

Data on the impact of alien plant species in Romania is still lacking and further studies are necessary. There is also a crucial need for coordinated institutional initiatives to enhance the effectiveness of alien species management at both national and local levels. Such efforts should encompass raising awareness and public engagement, harmonising legislation and enhancing the capacity of public institutions for invasive species management.

## Supplementary Material

94FEDF99-224B-5349-889F-044BAA265CAE 10.3897/BDJ.12.e119539.suppl1 Supplementary material 1 Alien plant species of Romania Data type Checklist Brief description Alien plant species of UE concern and alien plant species proposed as of concern for Romania (as December 2023). File: oo_973738.csv https://binary.pensoft.net/file/973738 Paulina Anastasiu, Iulia V. Miu, Athanasios A. Gavrilidis, Cristina Preda, Laurentiu Rozylowicz, Culita Sirbu, Adrian Oprea, Mihaela Urziceanu, Petronela Camen-Comanescu, Eugenia Nagoda, Daniyar Memedemin, Marius Barbos, Violeta Boruz, Alina Cislariu, Ioan Don, Marius Fagaras, Jozsef Pal Frink, Ioana Mihaela Georgescu, Ovidiu Ioan Haruta, Bogdan-Iuliu Hurdu, Attila Matis, Sretco Milanovici, Sorana Muncaciu, Alina-Georgeta Neacsu, Monica Neblea, Alma Lioara Nicolin, Mariana Niculescu, Silvia Oroian, Oliviu Grigore Pop, Daniel I. Radutoiu, Mihaela Samarghitan, Ioana Simion, Liliana Cristina Soare, Corina Steiu, Emilia Stoianov, Daniela Strat, Anna Szabo, Paul-Marian Szatmari, Corneliu Tanase, Marian D. Mirea, Nicolae Manta, Ioana M. Sirbu

## Figures and Tables

**Table 1.** Number of alien plant species per family and number of occurrence records included in the distribution database.

**Plant Family**	**Number of alien species**	**Number of occurrence records**
Asteraceae	66	34111
Fabaceae	23	16012
Amaranthaceae	25	6773
Poaceae	27	4004
Simaroubaceae	1	3908
Rosaceae	20	3721
Polygonaceae	6	3576
Solanaceae	18	3068
Moraceae	6	2818
Sapindaceae	5	2781
Cucurbitaceae	8	1951
Vitaceae	7	1662
Brassicaceae	15	1548
Elaeagnaceae	1	1101
Juglandaceae	4	1050
Onagraceae	9	949
Balsaminaceae	4	928
Oleaceae	4	784
Convolvulaceae	4	757
Anacardiaceae	2	707
Phytolaccaceae	2	689
Portulacaceae	2	671
Malvaceae	10	611
Oxalidaceae	4	552
Euphorbiaceae	8	496
Caprifoliaceae	7	443
Cannabaceae	4	397
Asphodelaceae	2	357
Salicaceae	6	324
Apocynaceae	1	271
Juncaceae	1	195
Plantaginaceae	3	194
Bignoniaceae	4	189
Paulowniaceae	1	127
Commelinaceae	2	78
Lamiaceae	9	67
Nyctaginaceae	1	52
Fagaceae	2	48
Ulmaceae	6	46
Apiaceae	3	44
Hydrocharitaceae	2	44
Crassulaceae	4	38
Papaveraceae	2	24
Scrophulariaceae	1	13
Araceae	1	12
Linderniaceae	1	12
Ranunculaceae	1	12
Berberidaceae	4	10
Hydrangeaceae	2	9
Acoraceae	3	8
Cyperaceae	3	8
Tamaricaceae	2	8
Salviniaceae	2	7
Asparagaceae	1	5
Cannaceae	3	5
Iridaceae	1	5
Molluginaceae	3	5
Caryophyllaceae	1	4
Violaceae	1	4
Amaryllidaceae	4	3
Boraginaceae	2	3
Cupressaceae	3	3
Rutaceae	2	3
Aizoaceae	2	2
Grossulariaceae	1	2
Heliotropiaceae	1	2
Saxifragaceae	1	2
Buxaceae	1	1
Cleomaceae	1	1
Linaceae	1	1
Menispermaceae	1	1
Musaceae	1	1
Nelumbonaceae	1	1
Pinaceae	1	1
Pteridaceae	1	1
Rhamnaceae	1	1
Rubiaceae	1	1
